# A Pupillometric Examination of Cognitive Control in Taxonomic and Thematic Semantic Memory

**DOI:** 10.5334/joc.56

**Published:** 2019-02-07

**Authors:** Jason Geller, Jon-Frederick Landrigan, Daniel Mirman

**Affiliations:** 1 University of Alabama at Birmingham, US; 2 Drexel University, US

**Keywords:** Semantics, Cognitive Control, Memory

## Abstract

Semantic cognition includes taxonomic and thematic relationships, as well as control systems to retrieve and manipulate semantic knowledge to suit specific tasks or contexts. A recent report (Thompson et al., 2017) suggested that retrieving thematic relationships (i.e., relations based on participation in the same event or scenarios) requires more effort or cognitive control, especially when the relevant relations are weak, than retrieving identity relations that are based on sensory-motor features. It is not clear whether the same contrast applies to the broader set of taxonomic relations, which are also based on shared sensory-motor features. In this study we tested cognitive control requirements of retrieving taxonomic and thematic knowledge using a physiological measure of cognitive effort: pupil dilation. Participants completed a semantic relatedness judgement task that manipulated semantic type (thematic vs. taxonomic) and relatedness strength (high vs. low) of word pairs. Cognitive control in the similarity task was examined using task-evoked pupillary responses (TEPRs), as well as standard behavioral measures (reaction times and accuracy). Compared with high-strength relations, low-strength semantic relations elicited larger TERPs, slower reaction times, and lower accuracy, consistent with higher control demands. Compared to thematic relations, taxonomic relations also elicited larger TERPs and slower reaction times, suggesting that retrieving taxonomic relations required more cognitive effort. Critically, our pupillometric data indicated that controlled processing was particularly important for low-strength taxonomic pairs rather than low-strength thematic pairs. These findings indicate that semantic control demands are primarily determined by relatedness strength, not whether the relationship is taxonomic or thematic.

## Introduction

Semantic knowledge is fundamental to nearly all aspects of cognition: it is how we know what to do with objects, it allows us to predict how different entities in the world will interact, and it gives meaning to language (McRae & Jones, 2013; Meteyard et. al., 2012; Tulving, 1972). Semantic knowledge is thought to be organized taxonomically, based on feature similarity or categorical membership (e.g., *dog* and *bear* are both animals; see Rogers & McClelland, 2004) and thematically, based on co-occurrence in the same events or scenarios (e.g., *dog* and *leash* both take part in the theme of walking; see Estes, Golonka, & Jones, 2011). A current debate concerns the cognitive and neural distinction between taxonomic and thematic semantic relationships. The dual-hub account (Mirman, Landrigan, & Britt, 2017; Schwartz et al., 2011) proposes distinct complementary taxonomic and thematic semantic memory systems that integrate distributed sensory-motor representations in different ways. The strongest evidence for separate systems comes from neuropsychological double dissociations showing that taxonomic and thematic knowledge can be impaired separately (for a systematic review of the dissociation of taxonomic and thematic systems see Mirman et al., 2017).

The controlled semantic cognition (CSC) framework (Lambon Ralph, Jefferies, Patterson, & Rogers, 2017) offers a different perspective on the organization of semantic knowledge. Within this framework, there is a single hub that integrates information from the distributed system of sensory-motor representations and a control system to manage context- and task-appropriate retrieval of semantic knowledge. According to this account, differences between taxonomic and thematic processing arise from accessibility or control requirements operating on a single semantic system (Davey et al., 2015; Thompson et al., 2017). In contrast, the dual-hub account suggests that cognitive control should be similar between taxonomic and thematic knowledge.

A recent study by Thompson et al. (2017; also see Davey et al., 2015) dissociated semantic control demands from task difficulty demands. In two experiments using a picture-to-word matching task, Thompson et al. (2017) compared a thematic matching task, with varied thematic relatedness strength (weak vs. strong), and an identity matching task, with varied specificity (i.e., object identity vs. superordinate), which affected task difficulty but not relatedness strength. Participants with semantic aphasia were more impaired at making thematic judgments than healthy controls were, particularly when matching weak thematic relationships. When neurologically intact participants performed the task under divided attention conditions, their thematic judgments (particularly weak thematic judgements) were more impaired than their object identity/superordinate judgments when performing a demanding secondary task (1-back) than when performing a minimally demanding secondary task (counting). Thompson et al. posited that the retrieval of thematic knowledge, especially if it is weak in relatedness strength, requires more control than identity judgments based on taxonomic specificity. Weak thematic associations probe unusual contexts and therefore have higher control demands than high thematic associations. Higher control demands are the result of inherent flexibility associated with weak thematic relations, which can be involved in many different events and scenarios so retrieval of the correct relation requires more control to shape retrieval. Identity relations are strongly constrained by shared sensory-motor features, so they require less support from semantic control. The identity-matching task varied in taxonomic specificity: participants had to match a photograph of an object (a dog) to its superordinate label (e.g., *animal*) or to a more specific term (e.g., *beagle*). This type of task does not require participants to retrieve information about the context. Thus, the identity matching task does not require the same control demands that judging weak and strong relationships does.

The Thompson et al. (2017) study leads to the question of whether the same contrast applies to other relationships that are constrained by shared sensory-motor features, namely, taxonomic relations. Non-identity taxonomic relationships such as *dog-wolf* are also based on shared features and do not depend on context, so they may similarly not require substantial semantic control. On the other hand, since the feature match is only partial, control may be required in order to focus on the relevant shared features rather than the non-shared features, especially when the feature overlap is low (i.e., weak taxonomic relations).

Here we investigate whether retrieving taxonomic and thematic relations differentially relies on control processes. We used a set of materials and methods that allowed a direct test of the taxonomic-thematic distinction, relationship strength, and cognitive control. Specifically, we used a semantic relatedness judgement task with a 2 × 2 factorial manipulation of semantic relation type (thematic vs. taxonomic) and relatedness strength (high vs. low) to examine whether weak thematic relationships (compared to strong thematic relationships) required more or less cognitive control than weak taxonomic relationships (compared to strong taxonomic relationships).

In order to make a relatedness judgment for a pair of words, the participant must retrieve the semantic relationship between two words, or fail to retrieve one and decide that no such relationship exists. When the relationship is weak, this retrieval will be particularly difficult and require more cognitive effort. The exertion of cognitive effort is closely linked with cognitive control mechanisms (Mulder, 1986). Two standard behavioral measures – reaction time and accuracy – are sensitive to cognitive effort, though they are also influenced by other factors (see, Balota & Yap, 2011). The task-evoked pupillary response (TEPR; Beatty, 1982) is a physiological measure that is also sensitive to cognitive effort (see Mathôt, 2018, for a review). Since the seminal work by Hess and Polt (1964) and Kahneman and Beatty (1966), researchers have reliably shown that pupil dilation correlates with cognitive or mental effort and can serve as an indirect marker of control within a task (van der Wel & van Steenbergen, 2018).

While our behavioral and physiological measures were predicted to converge, it is not uncommon for these measures to vary independently because pupil size is both more sensitive and more specific to cognitive effort (e.g., Geller et al., 2016; Kuchinsky et al., 2013; Papesh & Goldinger, 2012, Zekveld et al., 2010). For example, in a masked priming lexical decision task, Geller et al. (2016) found inhibitory priming differences in peak pupil size that were not present in the behavioral (RT and error rates) data. The pupillary response is recorded throughout the trial, thereby allowing a more fine-grained, temporal, analysis of task-evoked effort that is thought to be a purer index of cognitive effort (for more discussion see Geller et al., 2016).

Current evidence is mixed regarding whether taxonomic or thematic processing is more difficult. Across many tasks (e.g., priming and free sorting) judging thematic relationships appears to be easier than judging taxonomic relationships (Lawson, Chang, & Wills, 2017; Savic, Savic, & Kovic, 2017; but see Thompson et al., 2017). Using a single set of materials, Landrigan and Mirman (2018) found that two different tasks (triad and oddball) produced opposite patterns: thematic trials were easier in one task and taxonomic trials were easier in the other. This suggests that control might be a property of the task, and not inherent in the type of knowledge.

This study was designed to test whether semantic control is differentially recruited for retrieving feature-based (taxonomic) relations vs. event-based (thematic) relations. Both the dual-hub and CSC frameworks predict a main effect of relatedness strength: weak relations should elicit increased pupil dilation, take longer to process, and produce more errors, when compared to strong relations, across both relation types. Event-based thematic relations require focusing on event-relevant features whereas taxonomic relations are based on context-independent shared features, so taxonomic relations may require less control than thematic relations do. Within the CSC framework, the flexibility of thematic relations in comparison to object identity relations has been used to explain why individuals with semantic control deficits appear to be particularly impaired on thematic relations (e.g., Lambon Ralph et al., 2017; Thompson et al., 2017).

In contrast, the dual-hub framework makes no claim about taxonomic or thematic relations requiring more semantic control. Therefore, a main effect of semantic type (thematic relations recruit more control as denoted by increased pupil size, latencies, and error rates) would be inconsistent with the dual-hub framework, but could be accommodated by the CSC based on the idea that thematic relations are inherently more flexible. Similarly, the dual-hub framework makes no claim about an interaction between semantic type and relatedness strength, but the CSC could explain such an interaction if the inherent flexibility of thematic relations is particularly control-demanding when the relations are weak.

## Methods

### Preregistration

We preregistered the experiment before the data collection began on Open Science Framework (OSF; http://osf.io/grwtq). The preregistration contains a description of the research question, hypotheses, the sample size determination, and an analysis plan. We have performed data collection and analyses as described in the preregistration, unless otherwise stated. All data, materials, and scripts (i.e., pupil preprocessing and analysis) used can be found at http://osf.io/f6axd.

### Participants

Sixty participants from the University of Alabama at Birmingham participated for course credit. Given the uncertainty of the effect size in question, we preregistered a maximum participant sample size we would be willing to run (*N* = 60) in conjunction with a sequential analysis (see Lakens, 2017) performed at two evenly spaced time points (after 30 participants and 60 participants). The second analysis with the maximum sample size is reported here. All of the participants reported normal or corrected-to-normal vision and hearing. All participants reported no history of motor, cognitive, or neurological disorders.

### Materials

Word pairs were selected from a prior norming study (Landrigan and Mirman, 2016). In short, each pair was rated on a 7-point scale for its taxonomic similarity (sharing similar features and/or belonging to the same category) and its thematic relatedness (co-occurrence in common events). To be included in the current study, the pairs had to show a clear dichotomy in terms of the taxonomic and thematic relationships: taxonomic pairs had to have higher taxonomic similarity than thematic relatedness, and thematic pairs had to have higher thematic relatedness than taxonomic similarity. High strength pairs were selected to ensure relatively distinctive relations (taxonomic-thematic rating difference > 1.75) and weak non-dominant relation strength (mean rating ≤ 3.5). Low strength pairs were selected to exhibit a weaker dominant relationship (dominant relationship rating ≤ 4.5; taxonomic-thematic rating difference 0.5–1.75). These criteria ensured that weak pairs still exhibited a distinction in terms of their relationships but one that was weaker than the strong pairs both in terms of the distinction between taxonomic and thematic relatedness and weaker in terms of the dominant relationship rating. Because the non-dominant relationships were very weak for all word pairs, the relatedness judgment task was expected to be performed on the basis of the dominant relationship, which was substantially stronger for the high-strength pairs than for the low-strength pairs.

Filler trials were constructed using 64 unrelated word pairs. These pairs did not share similar features, did not belong to the same category, did not co-occur in common events, and were not associated according to the South Florida free association norms (Nelson, McEvoy, & Schreiber, 1998). All conditions were matched on word length in letters and phonemes, word frequency, orthographic neighborhood sizes, and imageability; see Table 1.

**Table 1:** Mean semantic and lexical properties as a function of semantic type and relatedness strength (standard deviations are provided in parentheses).

Characteristic	High-Taxonomic	Low-Taxonomic	High-Thematic	Low-Thematic	Filler	Comparison

Similarity rating	5.11 (.48)	3.61 (.345)	2.59 (.572)	2.19 (.749)		*t* = –10.53, *p* < .001^++^
Relatedness rating	3.05 (.386)	2.51 (.354)	4.87 (.537)	4.04 (.309)		*t* = 12.03, *p* < .001^++^
# of Letters	5.12 (1.74)	5.67 (1.67)	5.32 (1.43)	5.47 (1.74)	5.46 (1.68)	*ts* < .718
# of Syllables	1.53 (.563)	1.68 (.692)	1.59 (.657)	1.65 (.774)	1.63 (.665)	*ts* < 1.13
# of Phonemes	4.14 (1.63)	4.32 (1.34)	4.26 (1.37)	4.44 (1.46)	4.45 (1.53)	*ts* < .82
Ortho_N*	8.18 (8.91)	6.42 (7.01)	5.90 (6.97)	7.76 (8.66)	7.19 (8.07)	*ts* < .91
Phono_N*	15.6 (16.5)	13.39 (14.87)	12.4 (14.6)	12.5 (13.6)	13.9 (14.5)	*ts* < 1.57
Log(WF)*	2.63 (.665)	2.55 (.618)	2.71 (.625)	2.51 (.570)	2.75 (.646)	*ts* < 1.80
Log(CD)*	2.36 (.636)	2.31 (.570)	2.49 (.558)	2.58 (.683)	2.49 (.601)	*ts* < 1.51
Imageability*	583 (37.6)	584 (36.81)	569 (44.4)	582 (27.1)	578 (44.2)	*ts* < 1.51

*Note:* Word length in letters and number of phonemes obtained from the Speech & Hearing Lab Neighborhood Database at Washington University in St. Louis; stimuli were also matched on word frequency (*SUBTLEXUS*; Brysbaert & New, 2009), orthographic and phonological neighborhood size (*CLEARPOND*; Marian, Bartolotti, Chabal, & Shook, 2012), and imageability (*MRC Psycholinguistic Database*; Coltheart, 1981). *Imageability – ratings for 46 words not available; *Log (CD; contextual diversity) – ratings for 10 words not available; *Log (WF; word frequency) – ratings for 10 words not available; *Phono_N (number of phonographic neighbors) – ratings for 15 words not available; *Ortho_N (number of orthographic neighbors) – ratings for 15 words not available. ^++^the difference between taxonomic and thematic conditions, collapsed across relatedness strength.

In sum, each participant completed 128 trials in a 2 (semantic type: Taxonomic vs. Thematic) × 2 (semantic strength: High vs. Low) factorial design: 16 Low-Taxonomic pairs, 16 High-Taxonomic pairs, 16 Low-Thematic pairs, 16 High-Thematic pairs, and 64 filler pairs.

### Procedure

Participants were tested individually in a quiet room. The luminance level in the windowless laboratory was held constant at 230 lux. A chin and forehead rest maintained head position and viewing distance at 90 cm. The stimuli were presented in uppercase, 46-pt Courier New font on a constant gray background at 1920 × 1080 resolution on a 24-inch ASUS LCD monitor. Pupil data were continuously acquired monocularly (left eye) at 250 Hz, using a remote EyeLink 1000 plus eye tracker (SR Research). Response latencies were collected using a gamepad controller. The experiment was programmed using the Experiment Builder software provided by SR Research.

**Figure 1:**
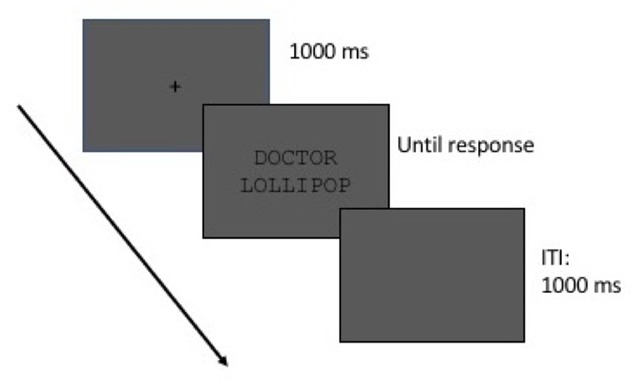
Schematic outline of a single experiment trial.

Following informed consent, a 9-point eye-tracker calibration and validation procedure was used and repeated until average deviations during validation were <0.50 degrees from target fixation. Participants were instructed to judge pairs of words as related or unrelated in meaning. The sequence of stimuli in each trial was the following: (1) a fixation cross (“+”) in the middle of the screen for 1,000 ms; (2) the fixation cross was replaced by a word above another word in the middle of the screen, which remained on the screen until the participant responded by pressing either the left button (“related”) or the right button (“unrelated”) on the gamepad; (3) a blank screen for 1,000 ms served as the inter-trail-interval (ITI) before the start of the next trial. A break was provided after each block of 16 trials (8 blocks). Each block contained filler pairs and either taxonomically or thematically related experimental pairs. Block order was counterbalanced. Before the experiment proper, participants completed 8 practice trials. The trial sequence is illustrated in Figure 1.

The pupil dilates reflexively to changes in luminance and eye position (Mathot, 2018; Papesh & Goldinger, 2012; Troscianko, 2003), which must be considered during stimulus generation and development of the procedural details (Papesh & Goldinger, 2012; Troscianko, 2003). To minimize these factors, all events in our experimental paradigm were presented in black font on a grey background in the center of the screen. Because pupil size is compared between conditions that are matched on luminance (i.e., low contrast, no color, constant lighting, lumnidistant stimuli) and eye position, minimal confound is present.

### Pupil Preprocessing

Preprocessing was accomplished using functions from the recently developed gazeR package (https://github.com/dmirman/gazer). The pupil data were first converted from arbitrary units to mm. Trials and participants with greater than 30% missing data were excluded (0.03% of trials; 1 participant replaced). Unrealistic pupil samples (i.e., pupil sizes smaller than 2 mm and greater than 8 mm) and pupil samples below and above 3 standard deviations of the mean diameter of each trial were removed (0.01%). Blinks, along with samples that occurred 100 ms before and 100 ms after the blink, were also coded as missing and linearly interpolated. After interpolation, artifacts that arose from quick changes in pupil size based on the median absolute deviation were removed (see Kret & Sjak-Shie, 2018). To smooth the pupil time course, a 5-point moving average was passed over the deblinked pupil data. Data from each trial were baseline-corrected using the median pupil diameter from the 500 ms prior to word-pair onset (during presentation of the fixation cross). A time window starting from word-pair onset (0 ms) to 4200 ms post-onset was selected for analysis, which deviates from our preregistered time-widow, as this range seemed to capture the entire rise and initial fall of the pupil response across conditions and individuals. To reduce computational cost given the large amount of data resulting from the eye-tracker output, the pupillary time course was epoched into 200 ms time bins.

### Growth Curve Analysis

Changes in pupil diameter were analyzed with a multi-level modeling technique known as growth curve analysis (GCA; Mirman, 2014; for recent applications to pupillometry see Kuchinsky et al., 2013; Winn, Edwards, & Litovsky, 2015). For the current analysis, GCA with third-order orthogonal polynomials was used to analyze the pupillary time course. In this model, the intercept refers to the average pupil size over the full time window; the linear time term refers to the slope of the pupillary time course (larger values indicating larger pupils size at the end of the time window than the beginning); the quadratic term refers to rate of acceleration or deceleration of the primary curve inflection point (more positive values indicate a flatter, more linear shape; more negative values indicate a steeper inverted-U shape); and finally the cubic term generally reflects the extent to which there is a secondary inflection point in the pupillary response (positive values indicates that the pupillary response had a more transient rise and fall whereas a negative value indicates it peaked later; Kuchinsky et al., 2013).

## Results

Per our preregistration plan, participants with less than 75% correct responses were replaced (4 participants), and word pairs with accuracy less than 60% were discarded (3 pairs: “STROLLER-SURFBOARD”, “LAGER-JUICE”, “COFFEE-NECTAR”). For our main effect predictions, we used an alpha level of 0.01 (one-tailed; *p*-values reflect this). For interactions, we used an alpha level of 0.01 (two-tailed). Linear mixed-effects models and GCA were implemented in R version 3.4.4 (R Core Team, 2018) using the lme4 package (Bates, Mächler, Bolker, & Walker, 2015) and p-values were derived using the lmerTest R package (Kuznetsova, Brockhoff, & Christensen, 2017).

### Behavioral Analysis

**Figure 2:**
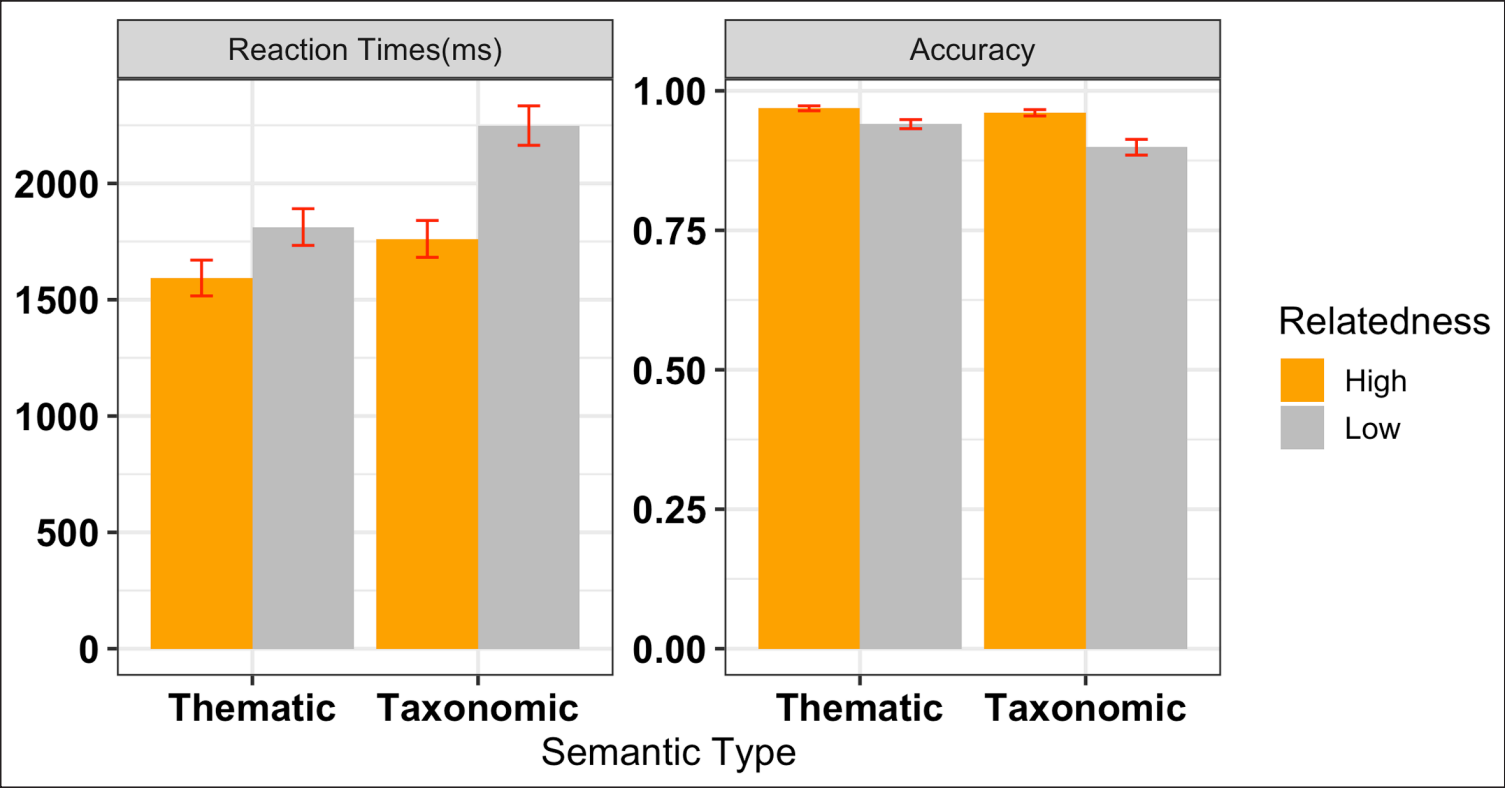
Mean Raw RTs (left) and Accuracy (right) as a function of Semantic Type and Relatedness Strength. Error bars reflect 95% Confidence Intervals (CIs). RT Model: -1000/rt ~ type * strength + (1 + type * strength | participant) + (1 | item); Accuracy Model: accuracy ~ type * strength + (1 + type | participant) + (1 | item).

Error trials (10%) and trials with very short correct RTs (<250 ms; 0 responses) were excluded from the latency analysis. Model-estimated RTs for correct responses and overall accuracy are presented in Figure 2.

For our reaction time analysis, inverse RT (–1000/rt)^1^ was used as the dependent variable to account for non-normality of the reaction time data. The semantic type and relatedness strength factors were both sum-coded (1, –1) to reflect the factorial design. We used a maximal random effect structure (Barr, Levy, Scheepers, & Tily, 2013). For our accuracy data, we used a generalized linear mixed model with the same fixed effects structure, but a reduced random effects structure, due to non-convergence. See Figure 2 for full model specifications.

Outliers with a standardized residual greater than 3 standard deviations were removed (1% of the data; see Britt, Ferrara, & Mirman, 2016) and the model was re-fit on the remaining latency data. The results indicated a statistically significant effect of semantic type: participants took longer to judge taxonomic pairs than thematic pairs, *Estimate* = –0.040, *SE* = 0.018, *p* = 0.015. There was also a significant effect of relatedness strength: high strength pairs were judged faster than low strength pairs, *Estimate* = –0.048, *SE* = 0.017, *p* = 0.004. Critically, semantic type did not interact with relatedness strength, *Estimate* = 0.014, *SE* = 0.017, *p* = 0.413. Although not specified in the preregistration, a Bayes factor (*BF*) derived from the Bayesian Information Criterion (see Wagenmakers, 2007) indicated that a model without the interaction term was strongly preferred (*BF* > 100; Jeffreys, 1961).

The analysis of the accuracy data did not show an effect of semantic type, *Estimate* = 0.202, *SE* = 0.168, *p* = 0.11. There was a significant effect of relatedness strength: accuracy was higher for high relatedness strength pairs than for low strength pairs, *Estimate* = 0.422, *SE* = 0.168, *p* = 0.003. Critically, semantic type did not interact with relatedness strength, *Estimate* = –0.124, *SE* = 0.162, *p* = 0.44. The *BF* indicated that a model with only semantic relatedness strength was preferred over the full model (*BF* = 52.16). This pattern of reaction time and accuracy data shows a clear effect of relatedness strength and mixed evidence suggesting possibly greater cognitive control recruitment for taxonomic relations, particularly low-strength taxonomic pairs. As described in the Introduction, pupil diameter offers a more sensitive and specific measure of cognitive effort, which is described in the next section.

### Pupillometry Analysis

**Figure 3:**
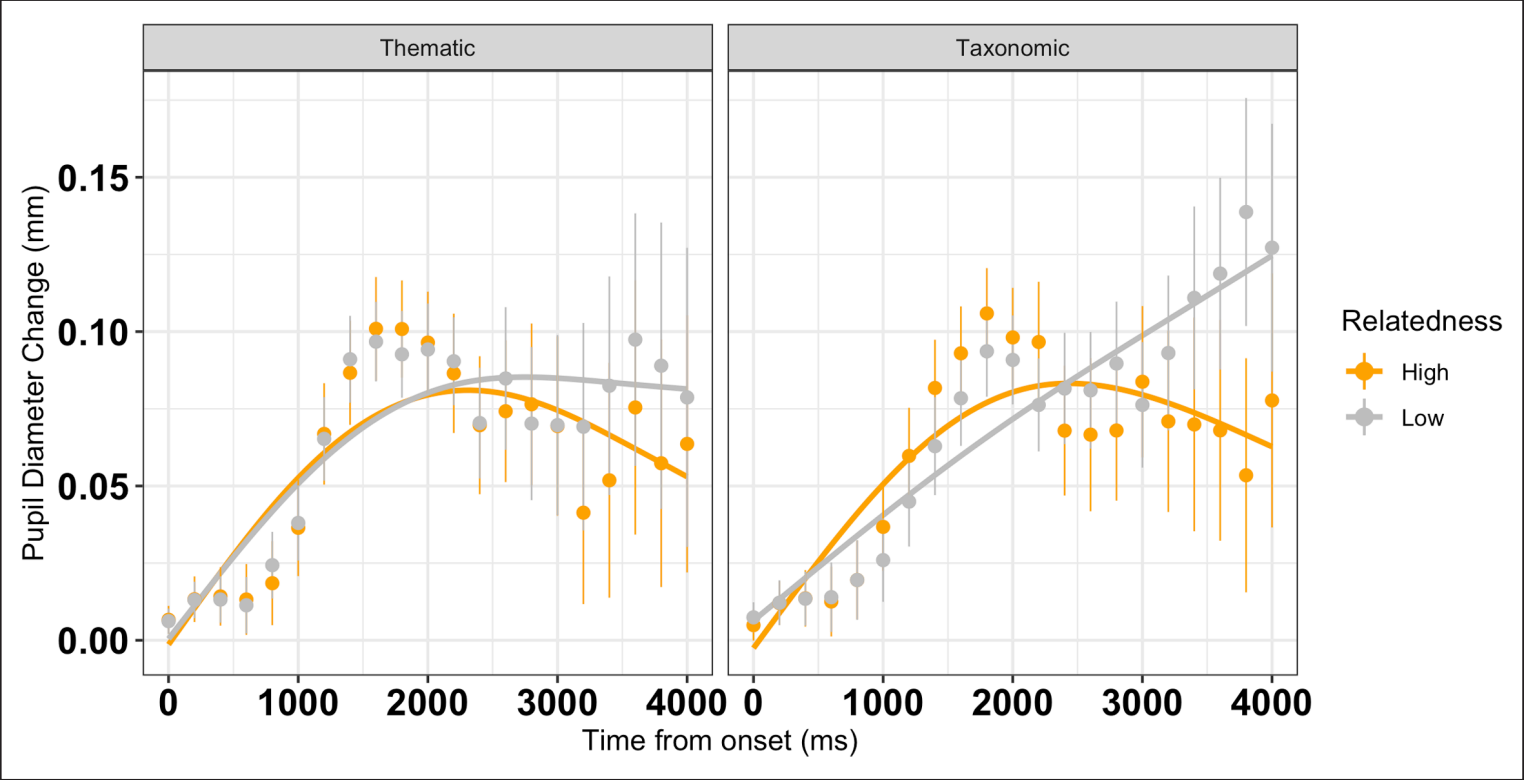
Baseline-corrected peak pupil dilation (dots, with SE vertical bars) overlaid with the GCA model fit (solid lines) as a function of semantic type and relatedness strength from stimulus onset until 4200 ms (200 ms time bins). Compared to low relatedness trials, on high relatedness trials, pupil diameter has a shallower slope and rises more quickly, then begins to decline. On low relatedness trials, the pupil dilation is steeper, slower, and longer-lasting, particularly for taxonomically related pairs.

The full results along with the model specification are presented in Table 2. The data and model fits are shown in Figure 3. The linear time term differed between taxonomic and thematic relations, *Estimate* = –0.01, *SE* = 0.007, *p* = 0.023; all other time terms (intercept, quadratic, and cubic polynomials) did not significantly differ between taxonomic and thematic relations, all *ts* < 1.01, *ps* > 0.5. That is, from the beginning of the trial to the end of the trial, the pupillary response increased more for taxonomic pairs than for thematic pairs. The effect of relatedness strength was significant on the linear, *Estimate* = –0.019, *SE* = 0.007, *p* < 0.001 and quadratic, *Estimate* = –0.014, *SE* = 0.007, *p* = .015, terms, but not on the cubic term, *Estimate* = 0.004, *SE* = 0.006, *p* = .500. This suggests that judging low strength pairs resulted in a steeper slope and shallower curvature (i.e., longer-lasting pupil response). The interaction between semantic type and relatedness strength differed on the linear, *Estimate* = 0.018, *SE* = 0.006, *p* = 0.006, and quadratic, *Estimate* = 0.018, *SE* = 0.007, *p* = 0.006, terms. For low relatedness taxonomic pairs, the pupil size continued to increase substantially, even in the late portion of the time window (e.g., more than 3000 ms after stimulus onset). In contrast, for the low relatedness thematic pairs, the pupil size was not substantially increasing by this point in the time course.

**Table 2:** Growth Curve Analysis Results for Baseline-Corrected Pupil Dilation. Values are the coefficient estimates with Standard Errors in parentheses.

	Overall		Semantic Type		Relatedness Strength		Type: Strength	

Intercept	0.06 (0.01)	***	–0.00 (0.00)		–0.00 (0.00)		0.00 (0.00)	
Linear	0.12 (0.03)	**	–0.01 (0.01)	*	–0.02 (0.01)	***	0.02 (0.01)	**
Quadratic	–0.07 (0.02)	**	–0.01 (0.01)		–0.01 (0.01)	**	0.02 (0.01)	**
Cubic	0.01 (0.02)		0.01 (0.01)		0.00 (0.01)		0.00 (0.01)	

***p < 0.001; **p < 0.01; *p < 0.05. Model: pupil ~ (poly1 + poly2 + poly3) * type * strength + ((poly1 + poly2 + poly3) + type * strength|subject).

## Discussion

The present experiment used a physiological (TEPR) measure of cognitive effort, in addition to RTs and accuracy, to test cognitive control recruitment during retrieval of high and low strength taxonomic and thematic semantic relations. Detecting taxonomic relationships resulted in longer reaction times and a steeper pupil dilation slope than detecting thematic relationships. For relatedness strength, behavioral and pupillometric analyses revealed (not surprisingly) that weak relations were more difficult than strong relations: they took longer to process, were more error-prone, and had steeper pupillary slopes. Our behavioral data did not show an interaction between semantic type and relatedness strength (Bayes factor analyses indicated main effect models were strongly preferred over interaction models). However, in our pupillometric analysis, we discovered an interaction between relation type and relatedness strength on linear and quadratic time terms. Pupil size increased more throughout the trial for low-relatedness taxonomic compared to low-relatedness thematic pairs. Also, in the late portion of the analysis time window, the pupil size continued to increase for low-relatedness taxonomic pairs more than for low-relatedness thematic pairs, suggesting ongoing cognitive effort.

Using a similarity relatedness judgment task, we found that judging taxonomic relations was more difficult than judging thematic relations, and weak relations required greater cognitive control than strong relations did, with greater cognitive control needed to judge low-strength taxonomic pairs. This pattern is opposite to the one observed by Thompson et al. (2017), in which thematic processing, especially for weakly related pairs, required more control than identity relations. The identity relation is a special case of taxonomic relations in which one item is an exemplar of the other item (e.g., *dog* – *animal*). Thompson et al. showed that such relations can vary in difficulty without necessarily differing in semantic control demands. They argued that this was because the relation is determined by feature overlap, making control demands minimal. The present study examined non-identity taxonomic relations (e.g., *dog* – *wolf*), which are also determined by shared features, but found that retrieving such relation *does* require semantic control. Indeed, retrieving weak taxonomic relations required more cognitive effort than retrieving weak thematic relations.

The reversal observed in our study compared to Thompson et al. (2017) suggests that task demands – not inherent controlled processing differences – determine whether taxonomic or thematic knowledge requires more cognitive control. In the current study, judging whether pairs are similar in meaning resulted in taxonomic relations being harder to judge than thematic relations. Conversely, thematic relations appear to be harder to detect than taxonomic relations across tasks involving multiple response options (e.g., picture-to-name matching; Thompson et al., 2017; Camel and Cactus Test; Hoffman, McClelland, & Lambon Ralph, 2018; oddball task; Landrigan & Mirman, 2017). One possible reason for this is that themes are more flexible, and when there are multiple response options, all possible relationships must be considered before selecting the dominate response. As previously argued by Landrigan and Mirman (2018), if control differences were an inherent property of taxonomic and thematic processing, they should be observed regardless of the task. Instead, across many studies, whether taxonomic or thematic relations are more difficult to detect seems to depend on the specific task.

This observation challenges claims that thematic relations are inherently more flexible than taxonomic relations and therefore require more control, and accounts of thematic semantic deficits as semantic control deficits. While we did not directly test the single- vs. dual-hub accounts, the present results are consistent with a dual-hub framework in which taxonomic and thematic semantic relations are represented by complementary semantic systems that integrate sensory-motor features in different ways and both of which engage cognitive control systems for retrieval of weak relations.

Although the data support an account that includes two independent semantic hubs, it does not necessarily exclude an account based on a single hub. A potential bridge between the CSC and the dual-hub frameworks is offered by a recent model (Hoffman et al., 2018) that builds on the hub-and-spoke origins of the CSC, but adds a distinction between feature-based concept identification and event-based prediction. This distinction is also a key aspect of the dual-hub view described by Mirman et al. (2017), who proposed that feature-based identification is the core of the taxonomic semantic system and event-based prediction is the core of the thematic semantic system. The Hoffman et al. model also includes a semantic control component that is important for retrieving context-appropriate semantic information and this “controlled retrieval” system appeared to be equally important for taxonomic and thematic relations (their Simulation 3), which is consistent with the dual-hub view and the behavioral and physiological data from the present experiment.

## Data Availability

All data, materials, and scripts used can be found at http://osf.io/f6axd.

## Footnotes

A model run with raw RTs showed the same effects.
